# Green Light‐Triggered Intraocular Drug Release for Intravenous Chemotherapy of Retinoblastoma

**DOI:** 10.1002/advs.202101754

**Published:** 2021-08-27

**Authors:** Kaiqi Long, Yang Yang, Wen Lv, Kuan Jiang, Yafei Li, Amy Cheuk Yin Lo, Wai Ching Lam, Changyou Zhan, Weiping Wang

**Affiliations:** ^1^ State Key Laboratory of Pharmaceutical Biotechnology Dr. Li Dak‐Sum Research Centre Department of Pharmacology and Pharmacy, Li Ka Shing Faculty of Medicine The University of Hong Kong Hong Kong SAR China; ^2^ Department of Pharmacology, School of Basic Medical Sciences Center of Medical Research and Innovation, Shanghai Pudong Hospital State Key Laboratory of Molecular Engineering of Polymers Fudan University Shanghai 200032 China; ^3^ Department of Ophthalmology, Li Ka Shing Faculty of Medicine The University of Hong Kong Hong Kong SAR China

**Keywords:** light‐triggered drug release, ocular drug delivery, retinoblastoma, self‐assembly, trigonal small molecules

## Abstract

Retinoblastoma is one of the most severe ocular diseases, of which current chemotherapy is limited to the repetitive intravitreal injections of chemotherapeutics. Systemic drug administration is a less invasive route; however, it is also less efficient for ocular drug delivery because of the existence of blood‐retinal barrier and systemic side effects. Here, a photoresponsive drug release system is reported, which is self‐assembled from photocleavable trigonal small molecules, to achieve light‐triggered intraocular drug accumulation. After intravenous injection of drug‐loaded nanocarriers, green light can trigger the disassembly of the nanocarriers in retinal blood vessels, which leads to intraocular drug release and accumulation to suppress retinoblastoma growth. This proof‐of‐concept study would advance the development of light‐triggered drug release systems for the intravenous treatment of eye diseases.

## Graphical abstract

A green light‐responsive nanocarrier is constructed based on the self‐assembly of photocleavable trigonal DTAEA molecule for achieving photo‐enhanced intraocular drug accumulation after intravenous injection of the drug‐loaded nanocarriers. The light‐triggered DOX release in orthotopic retinoblastoma exhibits significant anticancer efficacy and minimal side effects.

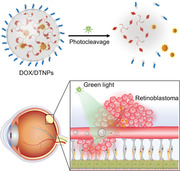

## 1 Introduction

Retinoblastoma (RB) is a severe eye disease affecting 1 among 15 000 infants every year and causing vision loss or even death.^[^ ^1^ ^]^ Drug administration through intravitreal injections could efficiently bypass the blood‐retinal barrier (BRB) and generate high local drug concentration at retinoblastoma sites, which has been considered as the most effective therapeutic route so far.^[^ ^2^ ^]^ However, such invasive drug administration route is unpleasant for patients and can lead to side effects such as ocular hemorrhage, endophthalmitis, retinal detachment, etc.^[^ ^3^ ^]^ Thus, drug administration routes in less invasive manner become more desirable for the treatment of retinoblastoma and other ocular diseases.^[^ ^4^, ^5^, ^6^ ^]^ Topical instillation and systemic administration of drugs provide alternative routes with improved safety compared with the intravitreal injection. However, low bioavailability of eye drops limits its clinical application for the treatment of retinoblastoma; unavoidable systemic toxicity and the inner BRB restrict the therapeutic efficacy of systemically administered chemotherapeutics.^[^ ^7^ ^]^ All these concerns are compelling the development of a novel and non‐invasive drug delivery strategy for the treatment of retinoblastoma to reduce the side effects and increase the therapeutic efficacy.

Over the decades, drug delivery systems have been developed to reduce side effects, prolong circulation time, precisely control the biodistribution, and increase the therapeutic efficacy of chemotherapeutics.^[^ ^8^ ^]^ By introducing external stimuli‐responsive moieties in drug delivery systems, the on‐demand release of drug molecules can be achieved after intravenous injection, resulting in controllable drug accumulation.^[^ ^9^, ^10^ ^]^ Among the various strategies, light activation has demonstrated to be a promising option, by which the release of bioactive molecules can be readily controlled with high spatiotemporal precision.^[^ ^11^, ^12^, ^13^, ^14^, ^15^ ^]^ Nevertheless, the currently used photocleavable groups in such systems usually require ultraviolet (UV) or blue light irradiation, which exhibited potential light‐induced toxicity and low penetrating ability.^[^ ^16^ ^]^ Compared with other tissues, the transparent vitreous body can be efficiently penetrated by light, which provides excellent potentials for light‐triggered drug delivery systems in the treatment of eye diseases. Recently, intraocularly injectable implants, including nano‐depots and hydrogels, were developed for UV/blue light‐triggered cargo release in the eye.^[^ ^17^, ^18^ ^]^ Moreover, 420 nm blue light was used to activate targeting ligands on nanocarriers to achieve light‐triggered targeting of the nanocarriers to the posterior eye.^[^ ^19^ ^]^ Light‐responsive ocular drug delivery has taken its first step in the treatment of diseases like choroidal neovascularization, but it has not been reported for the treatment of retinoblastoma.^[^ ^20^ ^]^

**Figure 1:**
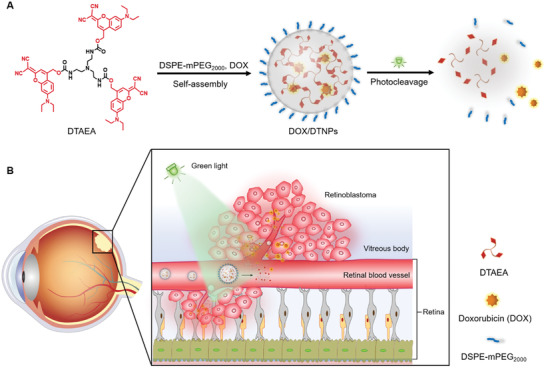
Schematic illustration of photo‐triggered chemotherapy of retinoblastoma. A) Self‐assembly, photodegradation, and drug release of the DOX‐loaded (DEAdcCM)_3_‐TAEA (DTAEA) nanocarrier; B) Proposed mechanism of photo‐enhanced drug accumulation in the eye.

Herein, we hypothesize that light‐triggered intraocular drug release after intravenous injection of drug‐loaded photoresponsive nanocarriers can efficiently result in drug accumulation at retinoblastoma and suppress the tumor growth. The strategy is mainly based on the efficient extravasation of the released hydrophobic drugs in the retina blood vessels to overcome the inner BRB that nanocarriers encounter. In this proof‐of‐concept study, we developed a green light‐responsive nanocarrier self‐assembled by clathrin‐like trigonal molecules with a simple yet photocleavable structure (**Figure** **1**A). The nanocarrier can rapidly respond to green light irradiation at 505 nm, which is less harmful than the commonly used UV/blue light.^[^ ^21^ ^]^ The distinctive clathrin‐like trigonal molecules can spontaneously co‐assemble with hydrophobic drugs in aqueous solutions via flash nanoprecipitation to form stable nanoparticles.^[^ ^22^, ^23^, ^24^ ^]^ The nanoparticles are able to release the encapsulated doxorubicin (DOX) upon the light irradiation. The release of DOX from the nanoparticles can be real‐time monitored based on the fluorescence resonance energy transfer (FRET) effect. The efficacy and safety of the nanoparticles for the treatment of retinoblastoma was demonstrated in an orthotopic retinoblastoma tumor model (Figure 1B). To our knowledge, this is the first demonstration of photo‐enhanced drug accumulation in the eye after intravenous injection of light‐triggered drug release systems.

## 2 Result and Discussion

The photocleavable trigonal molecule was synthesized through the route shown in Figure S1, Supporting Information, and the details are provided in Experimental Section, Supporting Information. Briefly, 7‐(diethylamino)‐4‐(hydroxymethyl)coumarin (DEACM), a commercially available photocleavable group that responds to violet‐blue light, was modified with dicyanomethylene group, and produced green light‐responsive dicyanomethylene‐coumarin (DEAdcCM) as the product. Because of the electron‐withdrawing effect of the dicyanomethylene group, the modification of carbonyl group with dicyanomethylene group results in a significantly red‐shifted absorption in the green‐light area (>500 nm) compared to the DEACM photocage.^[^ ^25^, ^26^ ^]^ The UV–vis spectra of both DEACM and DEAdcCM confirmed the red‐shifted absorption of DEAdcCM in the green‐light region compared to the DEACM group (Figure S2, Supporting Information). The DEAdcCM group was conjugated to a trigonal core molecule, tris(2‐aminoethyl)amine (TAEA), to give (DEAdcCM)_3_‐TAEA (DTAEA). After purification with a silica gel column, high performance liquid chromatography (HPLC), mass spectrometry, and nuclear magnetic resonance spectroscopy were performed to confirm the successful synthesis and purity of the final product (Figures S3–S5, Supporting Information).

**Figure 2:**
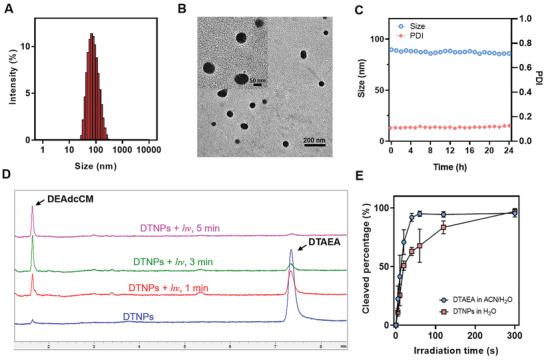
Preparation and characterization of DTNPs. A) Size distribution of DTAEA nanoparticles. B) Representative TEM images of DTAEA nanoparticles. C) Size and PDI of DTAEA nanoparticles in PBS at 37 °C within 24 h. D) HPLC elution curves of DTAEA nanoparticles before and after light irradiation in PBS solution. E) Photocleavage rate of DTAEA in 1:1 acetonitrile/water (v/v) solution and of DTAEA nanoparticles in water (*n* = 3).

Nanoparticles were prepared by one‐step nanoprecipitation method according to the reported protocol with minor modification.^[^ ^23^, ^24^ ^]^ DTAEA molecules self‐assembled into nanoparticles rapidly after adding the stock solution of DTAEA in DMSO into water. The *π*‐*π* stacking may play a vital role in constructing intermolecular interactions between dicyanomethylene‐coumarin groups, which facilitated the assembling process and stabilized the spherical nanostructure. Meanwhile, DSPE‐mPEG (10%, w/w to DTAEA) was co‐assembled with DTAEA for surface PEGylation, which can increase the water stability of the nanoparticles, reduce immune clearance, and prolong the circulation time after systemic administration.^[^ ^27^, ^28^, ^29^ ^]^ Dynamic light scattering (DLS) results showed the hydrodynamic size of 89.46 nm with a polydispersity index (PDI) of 0.14 (**Figure** **2**A). The prepared DSPE‐mPEG_2000_/DTAEA nanoparticles (abbreviated as DTNPs) were observed by transmission electron microscopy and identified as well‐dispersed spherical nanoparticles with the size of approximate 100 nm (Figure 2B), which was consistent with the DLS result. Moreover, the average zeta potential of DTNPs was measured as −25.10 mV (Figure S6, Supporting Information). The PEGylated and negatively charged surface may help DTNPs keep stable in aqueous solutions and reduce the clearance by the reticuloendothelial system.^[^ ^30^, ^31^ ^]^ Within 24 h at 37 °C, DTNPs in phosphate‐buffered saline (PBS) had a very slight change of size and their PDI stayed in the range of 0.10–0.15, indicating a good stability of the nanoparticles (Figure 2C). Moreover, the size of DTNPs kept stable in murine serum within 24 h under 37 °C, while marginally increased in the 10% fetal bovine serum (FBS)‐containing PBS. The size distribution curves at 0, 12, and 24 h were recorded and similar distributions without aggregation were observed (Figure S7, Supporting Information). In summary, DTNPs in PBS, 10% FBS‐containing PBS and murine serum are stable for at least 24 h under 37 °C.

The photocleavage mechanism of carbamate derivatives of coumarin photocleavable group was reported as a solvent‐assisted photo‐heterolysis process, in which one solvent‐trapped coumarin is produced from the carbamate molecule as the product.^[^ ^25^ ^]^ To quantitatively measure the photocleavage rate of DTAEA, DTAEA in the mixture solvent of acetonitrile and water (1:1, v/v) was irradiated with 505 nm green LED light. At predetermined time points (1, 3, and 5 min), the solution was analyzed by HPLC to determine the remained DTAEA and the released DEAdcCM (Figure 2D). More than 90% of DTAEA were cleaved under the irradiation (505 nm, 50 mW cm^−2^) within 1 min, revealing the rapid photocleavage process of the DTAEA molecule. The photocleavage of DTNPs in water took 5 min for the almost complete consumption of DTAEA, which was slower compared to free DTAEA in the mixture solvent (Figure 2E). The hydrophobic environment of the nanoparticles that retards the nucleophilic attack of carbocation intermediate by water molecules may be responsible for this result. Based on the photocleavage performance of DTNPs, the irradiation parameter was set to be 5 min with 505 nm green LED light at 50 mW cm^−2^.

As previously reported, the trigonal structure would be necessary for the self‐assembly of trigonal molecules.^[^ ^22^ ^]^ Thus, DTNPs would be destabilized following the photocleavage of DTAEA upon light irradiation. The size change of the nanoparticles upon light irradiation was also monitored by DLS (Figure S8, Supporting Information). Upon light irradiation, the hydrodynamic size increased, indicating the disruption of DTNPs and the formation of big aggregates from the hydrophobic cleavage products in the solution. These results demonstrated that the trigonal DTAEA molecules can self‐assemble into spherical nanoparticles, which exhibit photo‐triggered dissociation property due to the photocleavage of DTAEA.

**Figure 3:**
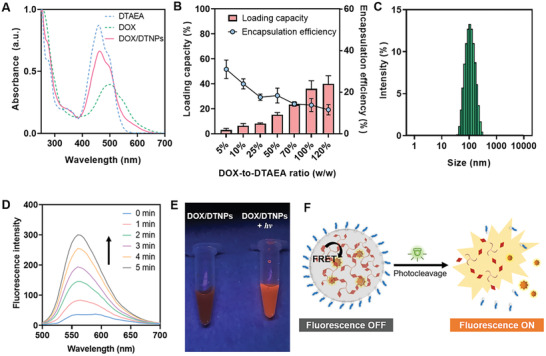
Characterization of DOX/DTNPs and the light triggered DOX release process. A) UV–vis spectra of DTAEA, DOX/DTNPs, and DOX. B) Encapsulation efficiency and loading capacity of DOX/DTNPs with different feeding ratios (DOX‐to‐DTAEA, w/w) (*n* = 3). C) Size distribution of DOX/DTNPs. D) Photo‐triggered fluorescence changes of DOX/DTNPs solution along with the irradiation time. E) Photos of DOX/DTNPs solutions before and after light irradiation (505 nm, 50 mW cm^−2^, 5 min) under 365 nm UV lamp. F) Scheme of the proposed mechanism of fluorescence monitoring of light‐triggered drug release from DOX/DTNPs.

DOX is a broad‐spectrum anticancer drug, showing outstanding anticancer efficacy in the treatment of retinoblastoma.^[^ ^32^ ^]^ Moreover, DOX is a typical lipophilic drug that has high permeability toward the BRB.^[^ ^33^, ^34^ ^]^ However, DOX administered by intravenous route was usually directly exposed to normal cells and rapidly eliminated in blood circulation, resulting in cytotoxicity in normal tissues and low intraocular concentration out of the therapeutic window.^[^ ^35^ ^]^ Herein, we chose DOX as the cargo to be encapsulated into DTNPs for light‐controlled drug delivery. Doxorubicin hydrochloride (DOX·HCl) was converted to free DOX by reacting with triethylamine overnight. Free DOX was dissolved in the stock solution of DTAEA and DSPE‐mPEG_2000_ for the flash nanoprecipitation in water. The three molecules could co‐assemble into the drug‐loaded nanoparticles (DOX/DTNPs) with mPEG_2000_ on the surface. The purification was then conducted by centrifugation, during which the nanoparticles are collected as the precipitate, and the unencapsulated drug and DMSO are removed in the supernatant. The UV–vis spectrum of the purified DOX/DTNPs showed broad absorption from 400 to 650 nm (**Figure** **3**A), which covered the absorption regions of DTAEA and DOX, demonstrating the successful encapsulation of DOX in DTNPs.

Subsequently, the loading capacity and encapsulation efficiency were determined and optimized by feeding different ratios of DOX to DTNPs during flash nanoprecipitation (Figure 3B). With the increased ratio of DOX to DTAEA, the loading capacity of DOX increased from 3.0% (1:20 DOX to DTAEA, w/w) to 36.0% (1:1 DOX to DTAEA, w/w). Further increasing DOX content would result in bulk aggregation and significant decrease of encapsulation efficiency, so we chose 1:1 as the final ratio of DOX to DTAEA for the preparation of DOX/DTNPs in the following studies. The encapsulation efficiency of DOX at this ratio was calculated to be 13.6%. Moreover, the size of DOX/DTNPs was determined as 99.04 nm, which did not change much compared to the cargo‐free DTNPs (Figure 3C).

To evaluate the light‐triggered drug release performance, the aqueous solution of DOX/DTNPs was irradiated by 505 nm light and analyzed by fluorescence spectroscopy. The fluorescence intensity of DOX/DTNPs (Ex. 480 nm) increased gradually with the increase of light irradiation time (Figure 3D,E). Such light‐triggered “fluorescence‐ON” phenomenon can be explained by the distance‐dependent FRET process between DTAEA and DOX (Figure 3F). When DOX was encapsulated into DTNPs, the fluorescence of DEAdcCM groups in DTAEA was quenched by DOX, since the distance of these two molecules was very close and the emission spectrum of DEAdcCM or DTAEA overlapped with the absorption spectrum of DOX (Figure S9, Supporting Information). Once DOX/DTNPs dissociated, the DOX molecules were released out of the resonance distance, resulting in the elimination of FRET and the recovery of the fluorescence of DEAdcCM. Based on the fluorescence change before and after irradiation, the release process of DOX from DOX/DTNPs can be real‐time monitored, which is useful for in situ imaging both in vitro and in vivo. The results also revealed that the dissociation of DTNPs and DOX release can be controlled by green‐light LED with a low irradiance at 50 mW cm^−2^.

To further confirm the light‐triggered cargo release from DTNPs, we fabricated Nile red (NR)‐loaded DTAEA nanoparticles (NR/DTNPs). NR is an environment‐sensitive dye, which is well known as a reporter of the formation and disruption of nanocomposites since its fluorescence can be enhanced in the hydrophobic environment while quenched in aqueous solutions.^[^ ^36^ ^]^ Thus, the spectrum changes of NR/DTNPs upon light irradiation can be monitored in a different manner compared to DOX/DTNPs (Figure S10A, Supporting Information). We found that the strong red fluorescence of NR/DTNPs decreased after light irradiation (Figure S10B, Supporting Information), which indicated the controlled release of NR from the nanoparticles upon the light irradiation.

**Figure 4:**
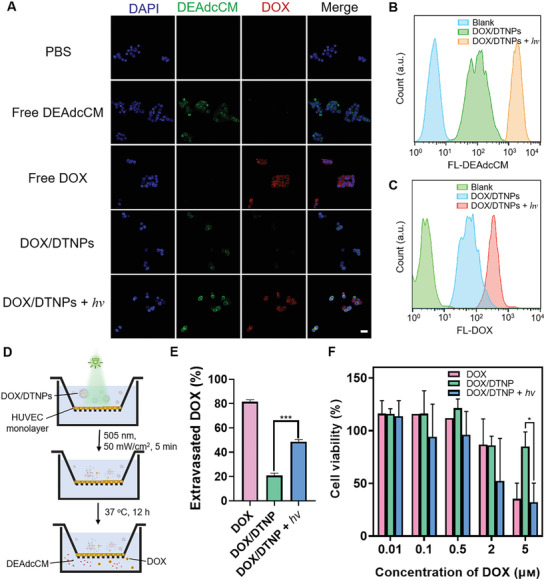
Cellular uptake, extravasation, and cytotoxicity of DOX/DTNPs. A) Representative fluorescent microscopic images of WERI‐Rb‐1 cellular uptake of DEAdcCM, DOX, or nanoparticles after 2 h incubation at equivalent DOX concentrations of 10 µм. B) Representative flow cytometry data of DEAdcCM fluorescence within WERI‐Rb‐1 cells incubated with DOX/DTNPs and treated with or without irradiation. C) Representative flow cytometry data of DOX fluorescence within WERI‐Rb‐1 cells incubated with DOX/DTNPs and treated with or without irradiation. D) Illustration of the Transwell‐based permeability study. E) In vitro extravasation of DOX across the HUVECs‐based BRB model (*n* = 3). F) Cytotoxicity of free DOX, DOX/DTNPs with and without light irradiation (505 nm, 50 mW cm^−2^, 5 min) on WERI‐Rb‐1 cells. The cells were treated with various formulations and incubated for 24 h before the cell viability measurement via CCK8 assay (*n* = 3). Data were shown as means ± SD. * *p* < 0.05, *** *p* < 0.005.

Encouraged by the performance of DTNPs in releasing loaded anticancer drugs upon light irradiation, we further investigated the cellular uptake of the released DOX by confocal laser scanning microscopy and flow cytometry. In this work, human umbilical vein endothelial cells (HUVECs) and human retinoblastoma (WERI‐Rb‐1) cells were employed to imitate the in vivo environment of retinal blood vessels and retinoblastoma tumors, respectively. The cells were treated with different formulations, including free DOX, DOX/DTNPs, and DOX/DTNPs + *hv*, at an equivalent DOX concentration of 10 µм. As shown in **Figure** **4**A and Figure S11, Supporting Information, both WERI‐Rb‐1 cells and HUVECs treated with DOX/DTNPs and green light irradiation showed stronger intensity in the red fluorescence channel of DOX compared to those cells treated with DOX/DTNPs without light irradiation. The result indicated the cellular uptake of DOX was increased when DOX/DTNPs were degraded and released DOX upon the light irradiation. The increased green fluorescence of DEAdcCM demonstrated the dissociation of DOX/DTNPs, which can be explained by the elimination of FRET between DEAdcCM and DOX. The 5 min irradiation group showed higher fluorescence intensity than the group irradiated for 2 min, further indicating that the uptake of DEAdcCM and DOX was increased by the light irradiation. Flow cytometry was utilized to quantitatively investigate the cellular uptake of DEAdcCM and DOX (Figure 4B,C). The WERI‐Rb‐1 cells treated with DOX/DTNPs and 5 min light irradiation showed both higher green (DEAdcCM) and red (DOX) fluorescence than that of non‐irradiated cells, which showed the same trend with the confocal imaging results.

To determine whether the light irradiation can control DOX extravasation across blood vessels, we constructed an in vitro inner BRB model (Figure 4D) based on previously reported Transwell method.^[^ ^37^ ^]^ The amount of DOX across through the endothelial monolayer with or without light irradiation was evaluated. Without light irradiation, only 20% of DOX was detected in the bottom chamber after 12 h incubation, indicating the DOX/DTNPs cannot extravasate efficiently (Figure 4E). However, the light irradiation achieved around 50% of DOX across the monolayer. The increased amount of extravasated DOX is attributed to the photo‐triggered DOX release from DOX/DTNPs, since free DOX has higher permeability across the endothelial monolayer than DOX/DTNPs due to its small size and hydrophobicity.

The cytotoxicity of DTNPs (with or without light irradiation) and the phototoxicity of green light irradiation were investigated by cell prohibition study via MTT cytotoxicity assay. HUVECs were plated in 96‐well plates and then treated with DTNPs at different concentrations. For the light irradiation group, the green light was applied for 5 min shortly after replacing the medium with the DTNPs‐containing medium. For the phototoxicity evaluation, the plated cells were irradiated for 0–30 min at 50 mW cm^−2^. We found no obvious cytotoxicity of cargo‐free DTNPs in the concentrations ranging from 0.9 to 180 µм with or without the light irradiation (Figure S12A, Supporting Information). The low‐irradiance green‐light irradiation (505 nm, 50 mW cm^−2^) is also harmless to the cells even when applied for up to 30 min (Figure S12B, Supporting Information). The cytotoxicity of light‐triggered DOX/DTNPs was evaluated via CCK8 assay with WERI‐Rb‐1 cells. As shown in Figure 4F, the viability of the cells that were treated with DOX/DTNPs and light irradiation significantly decreased, which resulted from the efficient drug release after the light irradiation. We observed less cytotoxicity in the cells treated with DOX/DTNPs, of which the cellular uptake is expected to be hindered due to the surface PEGylation and colloidal stability of the nanoparticles.

**Figure 5:**
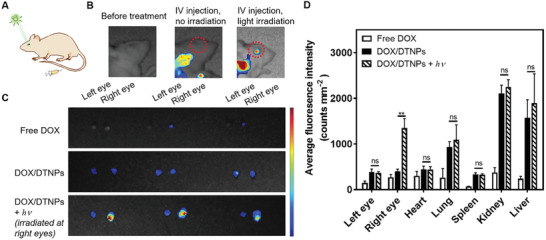
In vivo biodistribution of DOX/DTNPs. A) Illustration and B) representative IVIS fluorescent images of the mice 1 h post‐injection of DOX/DTNPs. For the irradiation group, the green light irradiation was conducted after intravenous injection. C) Representative fluorescent images of the excised mouse eyes 1 h after the injection of free DOX or DOX/DTNPs. D) Biodistribution of DOX and DEAdcCM determined by IVIS in different organs of the mice 1 h after the injection of DOX/DTNPs with or without light irradiation. The light irradiation (505 nm, 50 mW cm^−2^, 5 min) was performed post‐injection at the right eyes (means ± SD, *n* = 3; ** *p* < 0.01).

To validate that DOX/DTNPs can achieve light‐controlled drug release in the eye, an orthotopic retinoblastoma tumor model was established to evaluate the biodistribution and therapeutic efficacy. Generally, WERI‐Rb‐1 cells were injected slowly into the vitreous cavity of the right eyes of BALB/c nude mice for the tumor implantation. The tumor‐bearing mice were intravenously injected with DOX/DTNPs and then treated with or without light irradiation. The light irradiation was performed immediately post‐injection at the right eyes (**Figure** **5**A). The combined fluorescence of DEAdcCM and DOX imaged by the in vivo imaging system (IVIS), which were released from DOX/DTNPs, was observed in the right eyes after the light irradiation was performed. Minimal fluorescence signal was found in the left eyes of the irradiated group and in both eyes of the non‐irradiated group (Figure 5B,C). Moreover, to systematically evaluate the biodistribution of DOX, mice were euthanized after the above treatment, and their eyes (both sides), heart, lung, liver, spleen, and kidney were further excised for ex vivo fluorescence imaging by IVIS. Higher fluorescence intensity was observed in the right eyes of the irradiated group compared to others including the left eyes of the irradiated group and both eyes of the non‐irradiated group (Figure 5D and Figure S13, Supporting Information). The fluorescence intensity of the free DOX‐treated group is low in the organs and eyes, indicating the fast clearance of free DOX in vivo.^[^ ^38^ ^]^ These results indicated that intraocular drug release can only be achieved in the eyes treated with light irradiation following the intravenous injection of DOX/DTNPs. In addition, no significant difference was observed in heart, lung, liver, spleen, or kidney between the groups with and without light irradiation, revealing that the localized ocular illumination did not trigger drug release in other organs but only in the irradiated eyes. Such finding confirms that we successfully delivered drugs into orthotopic tumor‐bearing eyes by the light‐triggered release of DOX from DOX/DTNPs.

The safety of the nanoparticles and irradiation conditions was firstly studied in vivo. Murine fundus images of retinal blood vessels were collected after treating the eyes with DOX/DTNPs and DOX/DTNPs + *hv*, separately (Figure S14, Supporting Information). No obvious abnormalities, including vein occlusion and hemorrhage, were observed, indicating no noticeable adverse effects on retinal blood vessels. Moreover, both whole‐eye photography and hematoxylin and eosin (H&E) staining of cornea/retina slices did not display any abnormality after the DOX/DTNPs or DOX/DTNPs + *hv* treatments (Figure S15, Supporting Information). Thus, the irritation of eyes is minimal, since there was no structural damage caused by the nanoparticles and light treatment.

**Figure 6:**
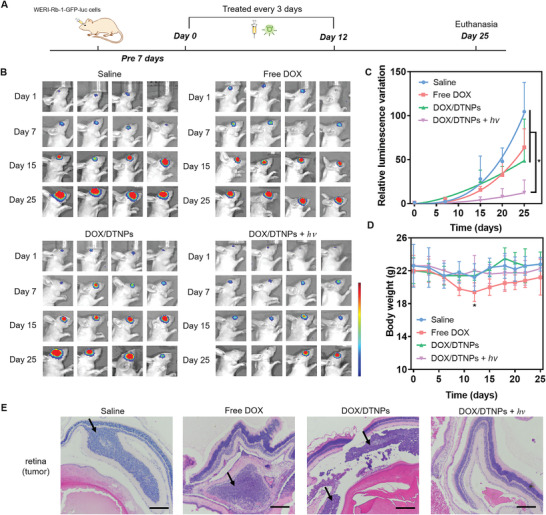
In vivo therapeutic effects of DOX/DTNPs. A) Illustration of the procedures for the treatment of retinoblastoma. B) In vivo bioluminescence images of the eyes in orthotopic WERI‐Rb‐1‐GFP‐luc tumor‐bearing BALB/c nude mice on day 1, 7, 15, and 25. C) Tumor growth curve presented by the intensity of bioluminescence of tumors after intravenous injection of various formulations. D) Body weight changes of the mice in each group. Data were shown as means ± SD (*n* = 4). * *p* < 0.05. E) H&E staining of the orthotopic retinoblastoma and retina tissue after different treatments. The scale bar is 200 µm.

To evaluate the in vivo therapeutic efficacy, WERI‐Rb‐1 cells were transfected with green fluorescent protein (GFP) and luciferase genes (abbreviated as WERI‐Rb‐1‐GFP‐luc) for in vivo tumor size monitoring. Seven days after the intraocular injection of WERI‐Rb‐1‐GFP‐luc cells at right eyes, the luminescence signals from the cells were determined by IVIS for in situ monitoring the growth of orthotopic retinoblastoma. The WERI‐Rb‐1‐GFP‐luc tumor‐bearing mice were randomly divided into 4 groups and intravenously injected with saline, free DOX, DOX/DTNPs, and DOX/DTNPs + *hv*, separately. The dosage of DOX administration was set as 5 mg kg^−1^ of body weight. Intravenous injection of the formulations was applied every three days from day 0 to day 12 for 5 times. After the injection, green‐light LED (505 nm, 50 mW cm^−2^, 5 min) was utilized to perform irradiation at the tumor‐bearing eyes of the group of DOX/DTNPs + *hv* (**Figure** **6**A). During the treatment period, bioluminescence from tumors was triggered by intraperitoneal injection of D‐luciferin and then detected by IVIS for tumor growth monitoring. As shown in Figure 6B, the mice treated with DOX/DTNPs and green‐light irradiation (the group of DOX/DTNPs + *hv*) showed a much slower increase of tumor bioluminescence intensity than those mice treated with other formulations (saline, free DOX, and DOX/DTNPs without light irradiation). To monitor the tumor growth process, tumor growth curves of different groups were obtained by setting the bioluminescence intensity on the day of the treatment (day 0) as the origin and determining the quantitative changes (compared to day 0) on the subsequent days (Figure 6C). On day 15, the group of DOX/DTNPs + *hv* showed obvious effect in tumor inhibition over other formulations. On day 25, the increase of bioluminescence intensity in the eyes treated with DOX/DTNPs + *hv* (7.3‐fold, compared to day 0) was significantly lower than those in the groups of saline (104.5‐fold, compared to day 0), free DOX (64.0‐fold, compared to day 0) and DOX/DTNPs (48.7‐fold, compared to day 0). It should be noted that two of the mice treated with DOX/DTNPs + *hv* showed negligible bioluminescence signal on day 25, indicating that their tumors were almost eliminated.

The body weight of the free DOX‐treated mice decreased about 10% during the drug administration period (day 0 to day 12) due to the side effects of the free chemotherapeutic drug (Figure 6D). On day 12, the body weight of the free DOX‐treated group displayed significant difference from other groups based on t‐test analysis (*p* < 0.05). For the groups treated with saline, DOX/DTNPs, and DOX/DTNPs + *hv*, no obvious loss in body weight was observed during the treatment, indicating unnoticeable systemic toxicity at least within 25 days. The histological patterns of tumor‐bearing retina and main organs were studied by H&E staining (Figure 6E and Figure S16, Supporting Information). The orthotopic retinoblastoma tumors distributed on the retina or in the vitreous body were observed in the slices excepted for the group treated with DOX/DTNPs and light, indicating the therapeutic effect of the treatment. Moreover, there was minimal histological alteration in the retina after receiving the treatment of DOX/DTNPs + *hv* for 25 days (Figure S17, Supporting Information), indicating the DOX/DTNPs and green light irradiation were compatible to the retina under the therapeutic regimen. Apart from the retina, main organs exhibited no apparent necrosis at the end of the study, suggesting minimal systemic toxicity of the intravenously injected DOX/DTNPs with light irradiation at the diseased eyes. Therefore, intravenous injection of DOX/DTNPs with light irradiation at the diseased eyes achieved both high therapeutic efficacy for retinoblastoma treatment and low systemic toxicity. The low systemic toxicity of DOX/DTNPs with light irradiation compared with free DOX is reasonably attributed to the stability of the PEGylated nanoparticles to minimize non‐specific drug release in normal tissues. DOX release triggered by the green light facilitated drug accumulation in the posterior segment of the eye, where the drug could take its effect for the retinoblastoma treatment.

## 3 Conclusion

The introduction of photocleavable groups into nanocarriers opens up a range of possibilities, in particular for drug delivery and imaging applications.^[^ ^39^ ^]^ We demonstrated the in situ monitoring of drug release from DOX/DTNPs based on the FRET process between DEAdcCM and DOX. The fluorescence changes upon light irradiation indicated the rapid dissociation of the drug‐loaded nanocarriers both in vitro and in vivo, which provides the possibilities of developing efficient imaging tools for monitoring payload release. Moreover, we developed the nanocarriers based on the self‐assemble of simple yet novel photoresponsive molecules. The trigonal structure of the molecules significantly promoted their self‐assembly to form nanoparticles, thereby improving the stability and modulating the in vivo fate of nanoassemblies.^[^ ^22^ ^]^ This study provides a strategy to design simple photoresponsive nanocarriers for controlled drug release.

For precise drug delivery with both high therapeutic efficacy and low side effects, the drug carriers need to remain intact during blood circulation but also be able to intelligently release the therapeutic payloads at diseased sites.^[^ ^40^ ^]^ To achieve this, stimuli‐responsive drug delivery that relies on the development of responsive smart materials has been confirmed to be an effective strategy. Light has widely used for triggering local photodynamic therapy, photothermal therapy, and on‐demand drug release.^[^ ^11^, ^41^ ^]^ Our results confirmed that the low‐irradiance green light (505 nm) has the ability to reach the retina area to activate the drug nanocarriers without noticeable phototoxicity or tissue damage. Compared to other tissues, the unique transparency of the vitreous makes it particularly beneficial for light‐triggered drug delivery strategy.

The development of therapeutic formulations for retinoblastoma therapy is usually restricted by the BRB. Systemic administration of free drugs is problematic due to the significant side effects associated with the large systemic doses needed. Our strategy utilized the light control to solve such problems. Before light irradiation, nanoparticles can circulate within blood vessels with minimal drug release and cause minimal side effects. Upon light irradiation to the eye, released free hydrophobic drugs are readily taken up by tumor endothelial cells or extravasate into tumor tissues by crossing the inner BRB,^[^ ^42^, ^43^ ^]^ increasing the drug accumulation in the retinoblastoma. The in vivo studies demonstrated the enhanced therapeutic efficacy with light irradiation.

In summary, we developed a photocleavable trigonal molecule DTAEA, which can self‐assemble into photoresponsive nanocarriers to achieve light‐triggered drug accumulation in the eye. Photo‐triggered DOX release in orthotopic retinoblastoma‐bearing mice achieved significant anticancer efficacy and exhibited minimal side effects. Besides ocular diseases, this light‐responsive drug delivery system can be applied for other diseases, where light can reach the diseased sites. Expanding the applications relies on the development of long‐wavelength light‐triggered drug delivery systems and efficient light delivery in the organism. We expect the systemic administration of triggerable nanocarriers can become a feasible strategy for delivering drugs to specific sites like eye, brain, and other barriered organs.

## Conflict of Interest

A PCT application was filed with No. PCT/CN2021/081262.

## Author Contributions

K.L. and Y.Y. contributed equally to this work. K.L., C.Z., and W.W. designed the experiments. K.L. and Y.Y. performed most of the experiments. K.L., Y.Y. and K.J. constructed the retinoblastoma model and performed the animal study. K.L., Y.Y. and W.W. wrote the manuscript, and all authors revised the manuscript.

## Supporting information

Supporting Information

## Data Availability

The data that support the findings of this study are available from the corresponding author upon reasonable request.
