# Correction to “Green Light‐Triggered Intraocular Drug Release for Intravenous Chemotherapy of Retinoblastoma”

**DOI:** 10.1002/advs.202416708

**Published:** 2025-04-26

K Long, Y Yang, W Lv, K Jiang, Y Li, A.C.Y Lo, W.C Lam, C Zhan, W Wang. Green Light‐Triggered Intraocular Drug Release for Intravenous Chemotherapy of Retinoblastoma. *Advanced Science* **2021**, 8(20), 2101754.

https://doi.org/10.1002/advs.202101754

In Figure 6B, the images of “Saline‐Day25‐Mouse4” and “DOX/DTNPs+hv‐Day15‐Mouse2” were mistakenly chosen during the layout of the images. The corrected Figure 6B is presented below. As the tumor growth curve (Figure 6C) was based on the correct quantitative data, the result and conclusion remain unchanged.


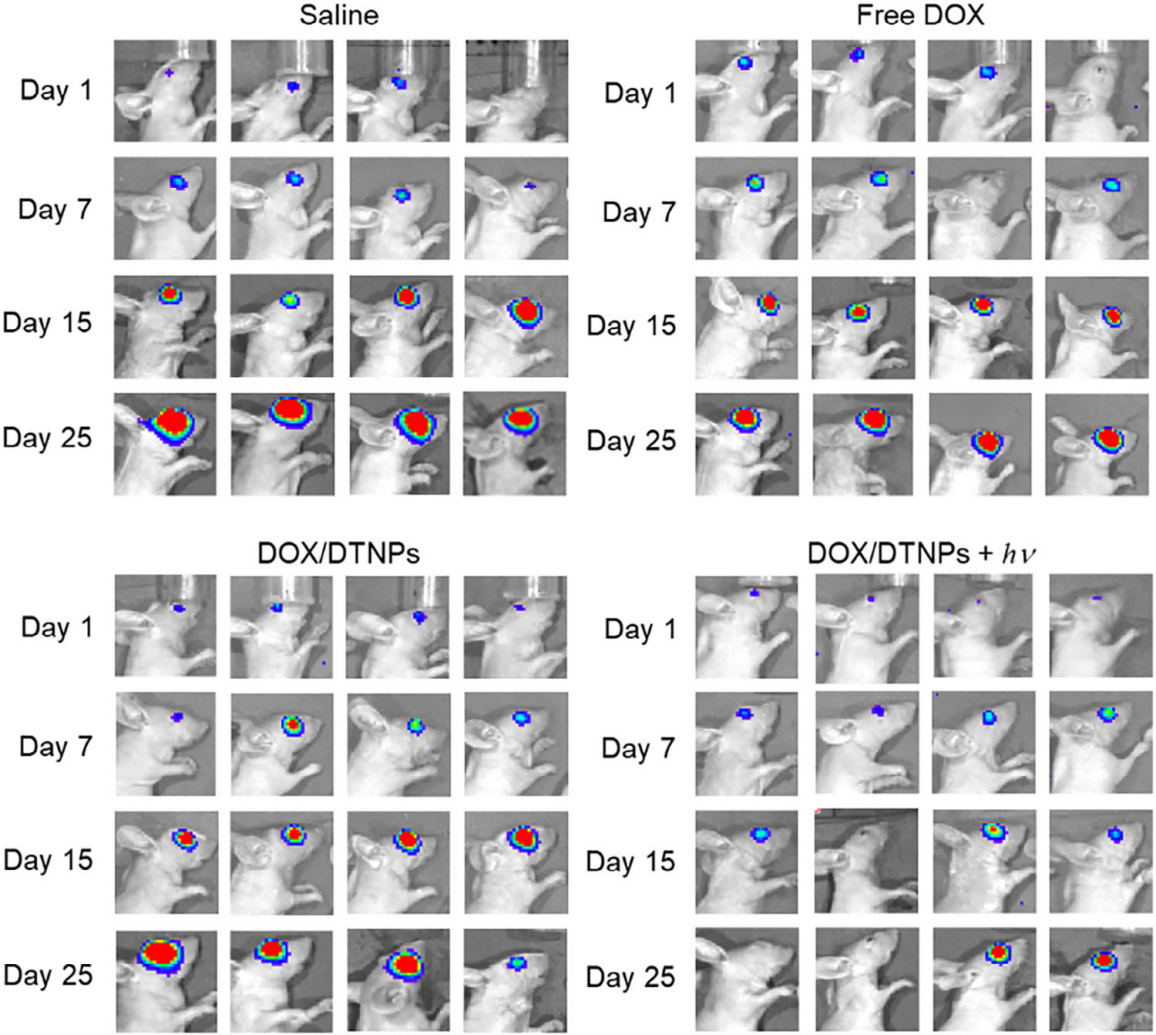


We apologize for this error.

