# Supplementary material for: Green Light‐Triggered Intraocular Drug Release for Intravenous Chemotherapy of Retinoblastoma
Source: Adv Sci (Weinh). 2021 Aug 27;8(20):2101754. doi: 10.1002/advs.202101754 (PMC8529428; doi:10.1002/advs.202101754)
Supplement: Supplementary file 1 — Supporting Information [file ADVS-8-2101754-s001.pdf]

© 2021 Wiley-VCH GmbH

ADVANCED SCIENCE
Open Access

Supporting Information

for *Adv. Sci.*, DOI: 10.1002/advs.202101754

Green light-triggered intraocular drug release for intravenous chemotherapy of retinoblastoma

Kaiqi Long,Yang Yang,Wen Lv, Kuan Jiang,Yafei Li, Amy Cheuk Yin Lo,Wai Ching Lam, Changyou Zhan,Weiping Wang**

Supporting Information

Green light-triggered intraocular drug release for intravenous chemotherapy of retinoblastoma

Kaiqi Long,Yang Yang,Wen Lv, Kuan Jiang,Yafei Li, Amy Cheuk Yin Lo,Wai Ching Lam, Changyou Zhan,Weiping Wang**

K. Long, Dr. W.Lv, Y. Li, Dr.W. Wang
State Key Laboratory of Pharmaceutical Biotechnology&Dr. Li Dak-Sum ResearchCentre&Department of Pharmacology and Pharmacy,Li Ka Shing Faculty of Medicine
The University of Hong Kong
Hong Kong SAR, China.
Email: wangwp@hku.hk

Y. Yang, Dr. K. Jiang, Prof. C. Zhan
Department of Pharmacology, School of Basic Medical Sciences & Center of MedicalResearch and Innovation, Shanghai Pudong Hospital & State Key Laboratory ofMolecular Engineering of Polymers
Fudan University
Shanghai, 200032, China.
Email: cyzhan@fudan.edu.cn

Dr. A. Lo, Prof. W. Lam
Department of Ophthalmology, Li Ka Shing Faculty of Medicine
The University of Hong Kong
Hong Kong SAR, China.

Keywords:light-triggered drug release, ocular drug delivery, retinoblastoma, self-assembly, trigonal small molecules

Table of Content

1. Experimental Section 3

Materials 3

Cell culture 3

Animals 4

Synthesis of $(DEAdcCM)_3$-TAEA (DTAEA) 4

Fabrication and characterization of DTNPs 7

Drug loading in DTNPs 7

Photocleavage of DTAEA molecules 8

Real-time fluorescence monitoring of drug release 9

Cellular uptake analysis 9

Cytotoxicity analysis 10

Extravasation of DOX across endothelial monolayer 11

In vivo biodistribution 12

Eye irritation and retinal reactions 12

Anti-tumor effects in orthotopic retinoblastoma tumor model 13

Statistical analysis 14

2. Figure. S1 to S17 14

1. Experimental Section

Materials

7-Diethylamino-4-hydroxymethylcoumarin (DEACM) was purchased from Bidepharm.N,N-diisopropylethylamine (DIPEA), tri-(2-aminoethyl) amine (TAEA), sodium sulphate anhydrous (Na_2SO_4), 4-(dimethylamino) pyridine (DMAP), N,N-dimethylformamide (DMF), dimethyl sulfoxide (DMSO) were obtained from Sigma-Aldrich. p-Nitrophenyl chloroformate was obtained from Alfa Aesar. Pyridine, anhydrous dichloromethane, triethylamine (TEA), hydrochloric acid (HCl), 3-(4,5-dimethyl-2-thiazolyl)-2,5-diphenyl-2-H-tetrazolium bromide (MTT) and other unlisted chemicals were obtained from J&K Co., Ltd. Acetonitrile (ACN), methanol, hexene, ethyl acetate, tetrahydrofuran and other solvents were obtained from Oriental Co., Ltd. DSPE-PEG_{2000} was supplied by Ponsure Biological Co., Ltd.

Cell Culture

Human retinoblastoma cells (WERI-Rb-1) and human umbilical vein endothelial cells (HUVECs) were purchased from Stem Cell Bank, Chinese Academy of Sciences. WERI-Rb-1 were cultured in RPMI-1640 (Gibco) supplemented with 10% FBS (Gibco) and 100 unitsmL^{-1} antibiotics (Penicillin-Streptomycin, Gibco) at 37 °C in a 5% CO_2 humidified atmosphere. HUVECs were cultivated in DMEM (Gibco) supplemented with 10% FBS and 100 unitsmL^{-1} antibiotics at 37 °C in a 5% CO_2

humidified atmosphere. The WERI-Rb-1-GFP-luc cells were transfected based on previously reported method without modification.[1]

Animals

BALB/c nude mice (male, 4 weeks, 20-22 g) were obtained from the Experimental Animal Center of Fudan Universityand maintained in SPF condition with access to food and water ad libitum.All the animal experiments were performed in compliance with the criteria of the National Regulation of China for Care and Use of Laboratory Animals.

Synthesis of (DEAdcCM)$_3$-TAEA (DTAEA)

Compound 2: DEACM (compound 1, 300 mg, 1.2 mmol) was dissolved in dry dichloromethane (DCM, 20 mL) in a duplex flask. Then acetic acid (83 µL, 1.44 mmol, 1.2 eq) and 4-(dimethylamino) pyridine (DMAP, 180 mg, 1.44 mmol, 1.2 eq) were added into the solution of DEACM. The mixture was cooled to 0°C and protected with nitrogen gas. 1,3-Dicyclohexylcarbodiimide (DCC, 300 mg, 1.44 mmol, 1.2 eq) was added slowly into the mixture. After stirring for 10 min at 0 ^{o}C, the mixture was warmed up to room temperature and stirred for 12 h in the dark. The mixture was then ten-fold diluted by DCM and washed with 1.2 M HCl and saturated solution of$NaHCO_3$separately for three times. The organic layer was collected and dried over $Na_2SO_4$and concentrated under vacuum. The residue was purified on a chromatography column by using 20:1 DCM/MeOH (v/v) to give compound 1 as yellow powder (Yield: 88.6%).

Compound 3: Compound 2 (311 mg, 1.1 mmol) and Lawesson's reagent (285 mg, 0.68 mmol, 0.62 eq) were dissolved in dry toluene (40 mL) and protected by nitrogen gas in the dark. The mixture was heated to 115°C and refluxed for 12 h. The solvent was removed by rotary evaporation and the residue was loaded into silica column directly. The product was eluted by DCM to give orange-yellow powder as the product (Yield: 77.6%).

Compound 4: Compound 3 (175mg, 0.57 mmol) and malononitrile (52 mg, 0.91 mmol) were dissolved in 4 mL ACN. The mixture was added into TEA (0.3 mL) and stirred for 2 h in the dark at room temperature. Thin layer chromatography was used to confirm the complete consumption of compound 3. Then $AgNO_3$ (221.8 mg, 1.3 mmol) was added and stirred for 2 h. After filtration, the solvent was removed by rotary evaporation. The residue was purified on a chromatography column by using 1:1 Hexene/DCM (v/v) to give compound 4 as orange-red powder (Yield: 72.4%).

Compound 5: Compound 4 (140mg, 0.41 mmol) was dissolved in absolute ethanol (50 mL). Aqueous HCl (37%, 3.36 mL, 0.04 mol) was added slowly and the mixture was refluxed at 85°C for 16 h in the dark under nitrogen gas. The solvent was removed under reduced pressure and purified on a chromatography column by using DCM to give compound 5 as orange powder (Yield: 88.2%).

Compound 6: Compound 5 (108 mg, 0.37 mmol) was dissolved in 10 mL dry DCM. DIPEA (0.71 mL, 4.1 mmol) was added, and the mixture was cooled to 0°C in the dark. After stirring for 15 min, the solution of 4-nitrophenyl chloroformate (0.83 g, 4.1 mmol) in 5 mL dry DCM was dropwise added into the mixture. The resulting mixture was allowed to warm to room temperature and stirred for 6 h. The mixture was washed by 0.01 M HCl solution (100 mL x 2). The organic layer was collected and evaporated under reduced pressure. The residue was purified on a chromatography column by using 20:1 DCM/ethyl acetate (v/v) to give compound 6 as red powder (Yield: 90.8%).

Compound 7 (DTAEA): Compound 6 (153 mg, 0.33 mmol) was dissolved in 1.5 mL dry DCM under nitrogen gas and cooled to 0°C. DIPEA (105 μL, 0.6 mmol) was added and stirred for 15 min. The solution of TAEA (15 μL, 0.1 mmol) in 1 mL dry DCM was slowly added into the above mixture at 0°C. The resulting mixture was allowed to warm to room temperature. After stirring for about 1 h, a small amount of precipitation can be observed. Then, more DIPEA (105 μL, 0.6 mmol) was added and the mixture was stirred overnight. Thin layer chromatography was used to confirm the complete consumption of compound 6. Then the residue was evaporated under reduced pressure and loaded on a chromatography column. DCM/MeOH (0% to 4%) was used to elute the final product as orange-yellow powder (Yield: 63.4%).

Fabrication and characterization of DTNPs

The nanoparticles were fabricated by flash nanoprecipitation method that was reported previouslywith modifications [19, 20]. Briefly, DTAEA (10 mgmL^{-1}) and DSPE-mPEG$_{2000}$ (20 mgmL^{-1}) were dissolved in DMSO separately and then mixed at the weight ratio of 10:1 to form a stock solution. The stork solution (5 μL) was then added into 200 μL of filtrated water with vortexing. The resulting orange solution was further sonicated for 5 min at room temperature to give uniform nanoparticles. Then the solution was purified via a differential centrifugation method by ultrahigh-speed low-temperature centrifugate (ST 8R, Thermo Fisher Scientific, Waltham, MA, USA). The solution of nanoparticles was first centrifuged at 3000×g for 10 min and the precipitation was removed, the process was repeated for three times or more until no precipitate was observed. The supernatant was then collected and further centrifuged at 30000×g for 20 min. The precipitated nanoparticles were washed for three times or more with water. The nanoparticles were finally collected as precipitates and dispersed into water or PBS. The concentration of DTAEA in DTNPs was determined by HPLC. DTNPs were imaged by Philips CM100 transmission electron microscope. The size distribution and zeta potential of DTNPs were measured by dynamic light scattering (ZetasizerNano ZS90, Malven Instrument, southborough, MA, USA).

Drug loading in DTNPs

DOX can be encapsulated into DTNPs via co-assembly method during the flash nanoprecipitation of nanoparticles.[2,3] Hydrophobic DOX was obtained by the

conversion of commercial DOX hydrochloride with addition of TEA. Briefly, 10 mg DOX hydrochloride was dissolved in 1 mL DMSO and 2-fold molar of TEA was added. The solution was kept stirring overnight. Subsequently, the solution was mixed with the stock solution of DTAEA and DSPE-mPEG$_{2000}$ at different weight ratios (from 5% to 120%, w/w of DOX/DTAEA), in which the amount of DSPE-mPEG was 10% (weight ratio) of DTAEA. Drug-loaded nanoparticles (DOX/DTNPs) were fabricated and purified based on the fabrication protocol of DTNPs. The drug loading at different drug-to-material ratios were evaluated. To quantitatively determine the drug loading, the purified DOX/DTNPs was diluted and analysed by HPLC. The concentration of DTAEA and DOX in the nanoparticle solution was calculated based on a concentration-peak area standard curve. Drug loading capacity and encapsulation efficiency were calculated as follow:

$$\text{Loading capacity } (\%) = \frac{\text{weight of loaded drug}}{\text{weight of nanoparticles}} \times 100\%$$

$$\text{Encapsulation efficiency}(\%) = \frac{\text{weight of loaded drug}}{\text{weight of feeded drug}} \times 100\%$$

Photocleavage of DTAEA molecules

Newly prepared DTNPs was collected and dispersed into PBS solution (10 mM, pH 7.4). The solution was added into a 1.5 mL vial and irradiated by green light (505 nm, 50 mWcm^{-2}, 0-5 min). Samples at different time points (irradiated for 0 min, 1 min, 3 min, and 5 min) were collected and directly analyzed by HPLC (C18 column, Poroshell 120, EC-C18, 2.7 μm). The mobile phase consisted of ACN and H_2O with 0.1% TFA. The

percentage of ACN was increased from 30% to 100% within 10 min with a flow rate at 1.5 $mLmin^{-1}$.

Real-time fluorescence monitoring of drug release

Newly prepared DTNPs was collected and dispersed into PBS solution (10 mᴍ, pH = 7.4). The solution was put into a cuvette and irradiated by green light (505 nm, 50 $mWcm^{-2}$, 0-5 min). At different time points (0, 1, 2, 3, 4, and 5 min), the fluorescence was measured by a spectrometer (SpectraMax® M4, Molecular Devices LLC, San Jose, CA) without any dilution. For the Nile Red-loaded nanoparticles (NR/DTNPs), the excitation wavelength was set as 562 nm.

Cellular uptake analysis

For confocal imaging, HUVECs or WERI-Rb-1 cells were cultured in complete medium and plated in confocal plates (Corning, 200350, Cell Culture-Treated) at a density of 10000 cells per well.Formulations including PBS, free DEAdcCM, free DOX, DTNPs, DOX/DTNPs at several concentration gradients were added into the medium.For the light-irradiated group, green-light LED (Mightex Systems., CA, USA) (505 nm, 50 $mWcm^{-2}$) irradiated on the bottom of the cell plate for 5 min after adding formulation solutions. After 4 h, the cells were washed 3 times with 10 mᴍPBS (pH 7.4) at 37 °C. Cells were observed under a confocal laser scanning microscope (LSM 780, Carl Zeiss, Germany).

For flow cytometry, WERI-Rb-1 cells were cultured in complete medium and seeded in 12-well plates at a density of 20000 cells per well. The cells were cultured for 24 h and then added with different formulations including PBS, free DOX, DTNPs, and DOX/DTNPs. For the light-irradiated group, light irradiation (505 nm, 50 $mWcm^{-2}$) was performed for 5 min. After 4 h, the cells were washed 3 times with 10 mM PBS (pH 7.4) and the mean fluorescence intensities were tested by a flow cytometer (BD FACSCanto™ II Cell Analyzer, United States). Specifically, the fluorescence of DEAdcCM was detected by FL1 channel (green fluorescence, ex. 488 nm, em. 530 nm) and the fluorescence of DOX was detected by FL3 channel (red fluorescence, ex. 515 nm, em. 661 nm).

Cytotoxicity analysis

Cell viabilities of HUVECs were determined by MTT assay. HUVECs were cultured in 96-well plates at a primary density of 5000 cells per well and incubated for 24 h before adding the formulations. The growth medium was replaced with the nanoparticle solutions at different concentrations. For the light-irradiated group, the light irradiation (505 nm, 50 $mWcm^{-2}$) was performed for 5 min. MTT solution (10 μL per well) was added after 24 h of incubation and the OD490, OD570 and OD630 values were measured for the calculation of cell viability.

For WERI-Rb-1 cells, cell viabilities were evaluated by CCK8 assay. WERI-Rb-1 cells were plated in 24-well plates at a density of 50000 cells per well. Various formulations were added into the culture medium and continued to incubate for 24 h. Cells were then

collected by centrifugation (1000 g, 5 min) and washed with PBS for 3 times. The collected cells were re-dispersed in a new 24-well plate with fresh culture medium. CCK8 solutions (100 μL per well) were added and OD450 values were measured after 4 h of incubation for the calculation of cell viability.

Extravasation of DOX across endothelial monolayer

The *in vitro* inner BRB model was constructed based on the previously reported Transwell® method.[4] HUVECs were seeded onto the upper side of the 6.5 mm Transwell® with 0.4 μm pore polytetrafluoroethylene membrane inserts coated with collagen type I (Thermo Fisher Scientific, Waltham, MA, USA) at a density of 2×10^5 cells per cm^2. The inserts were placed in 24-well plates containing complete medium. The medium in the plates with inserts was changed every day.

Trans-endothelial electrical resistance (TEER) values were obtained via epithelial voltammeter (EVOM2, World Precision Instrument co. ltd. Sarasota, FL, USA) to confirm the successful construction of endothelial monolayers.

Endothelial monolayers with TEER over 100 $\Omega \cdot cm^2$ were chosen for the experiments. The apical side of barriers was incubated with free DOX or DOX/DTNPs at the equivalent DOX concentration of 5 μM. Light irradiation (505 nm, 50 $mWcm^{-2}$, 5 min) was applied on the top of the Transwell® inserts. The DOX concentration at the bottom chamber was then measured by HPLC after 12 h and the extravasated percentage of DOX was calculated.

In vivo biodistribution

Orthotopic retinoblastoma-bearing mice were established as the reported method.[1] Briefly, male BALB/c nude mice were anesthetized with 40 mgkg^{-1} pentobarbital sodium by intraperitoneal injection. Ocular local anesthesia was conducted by topical application of 0.4% oxybuprocaine hydrochloride, followed by 0.5% tropicamide to dilate the pupil. The retinoblastoma cells were suspended in PBS at a density of 10000 cells perμL. Two μL of the cell solution was loaded into the micro syringe (33 G, Hamilton™, Thermo Fisher Scientific Inc., Waltham, MA, USA), which was slowly injected into the bottom of the vitreous cavity of the right eye. After removing the micro syringe, 0.5% chloramphenicol was topically administrated. The mice were further kept in SPF condition for 5-7 days until vitreous turbidities were observed.

Three groups (n = 3) of the tumor-bearing mice were intravenously injected with free DOX andDOX/DTNPs (5 mgkg^{-1} on the DOX basis) with or without the light irradiation (505 nm, 50 mWcm^{-2}, 5 min), separately.The light irradiation was performed post-injection at the tumor-bearing right eyes. The combined fluorescence of DEAdcCM and DOX from DOX/DTNPs was measured by IVIS (CailperPerkinElemer, United States). The mice were then euthanized. Organs including heart, lung, liver, spleen, kidney, as well as the eyes, were excised and washed with PBS for 3 times before the *ex vivo* fluorescence imaging by IVIS.

Eye irritation and retinal reactions

To evaluate the eye irritation of the formulations and light irradiation, BALB/c nude mice were intravenously injected with DOX/DTNPs or DOX/DTNPs + *hv*, separately.

The dose of nanoparticles was set as 5 mgkg^{-1} on the basis of DOX, and the light irradiation was set at 50mWcm^{-2}, 5 min. The whole-eye photographsand fundus images of retinal blood vessels of the eyes were collected 1 h post-treatment with a Phoenix Micron IV retinal imaging microscope (Phoenix Technology Group, LLC.CA, USA). The mice were then euthanized, and the cornea and retina were excised and stained by H&E assay for histological analysis.

Anti-tumor effects in orthotopic retinoblastoma tumor model

The bioluminescence monitorable and orthotopic retinoblastoma-bearing mice were established by using WERI-Rb-1-GFP-luc cells during tumor implantation. The intraocular bioluminescence at the right eyes of the mice was detected 1 week aftertumor implantation. To induce bioluminescence, tumor-bearing mice were intraperitoneally injected with 150 mgkg^{-1} D-luciferin and anesthetized with isoflurane before IVIS imaging. Sixteen tumor-bearing mice with approximative bioluminescence intensities were selected and randomly divided into four groups (n = 4) and intravenously injected with saline, free DOX, DOX/DTNPs and DOX/DTNPs+ *hv*, separately. The dosage of drug administration was precisely controlled based on the body weight of mice and set as 5 mgkg^{-1} of body weight. The injection of the formulations as well as the light irradiation after the injection were applied every three daysfrom day 0 to day 12 for 5 times . The mice were continued to be fed in a dark room and monitored until day 25. *In vivo* imaging of bioluminescence was carried out on day 0, 7, 15, 20, 25. After the treatment, the mice were euthanized on day 25. The treated

eyes were fixed in 4% paraformaldehyde and stained with hematoxylin-eosin (H&E) for further observation and histology analysis.

Statistical analysis

All experiments were conducted three times or more independently. Data were presented as the mean ± standard deviation (S. D.). The one-way ANOVA method and Independent-samples t-test were adopted to determine the statistical significance of differences using GraphPad Prism 8.0.2 software. The criterion for statistical significance was taken as $^{*}p < 0.05$, $^{**}p < 0.01$ and $^{***}p < 0.001$.

2. Figure. S1 to S17

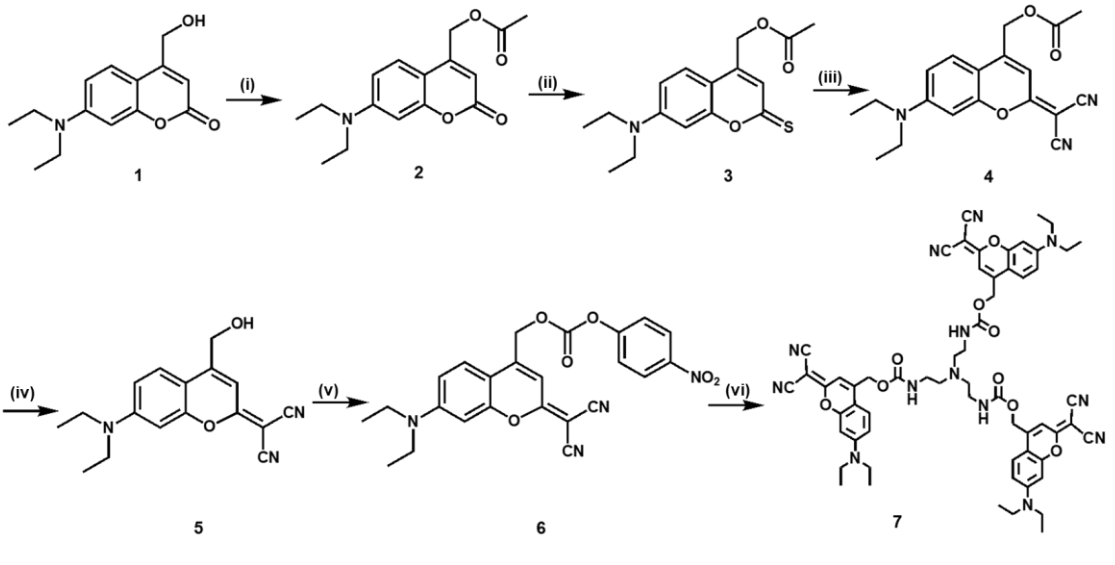

Figure S1. Synthesis route of the DTAEA molecule. (i) Acetic acid, DCC, DMAP; in DCM, 12 h; (ii) Lawesson’s reagent; in toluene, 115 ^{o}C, 12h; (iii) $CH_2(CN)_2$, TEA, $AgNO_3$; in ACN, r.t., 2 h; (iv) HCl; in EtOH, 85 ^{o}C, 16 h; (v) 4-nitrophenyl chloroformate, DIPEA; in DCM, 0 ^{o}C, then r.t., 6 h; (vi) TAEA, DIPEA; in DCM, r.t., overnight.

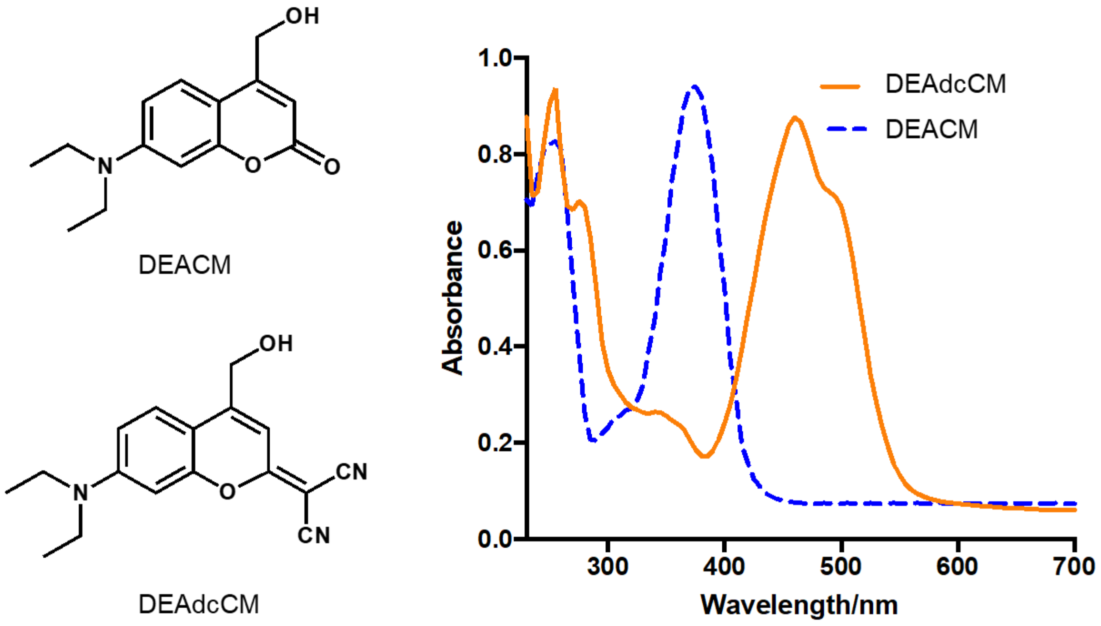

Figure S2. Molecular structure and UV-vis absorption spectra of two photocleavable groups, DEACM and DEAdcCM.

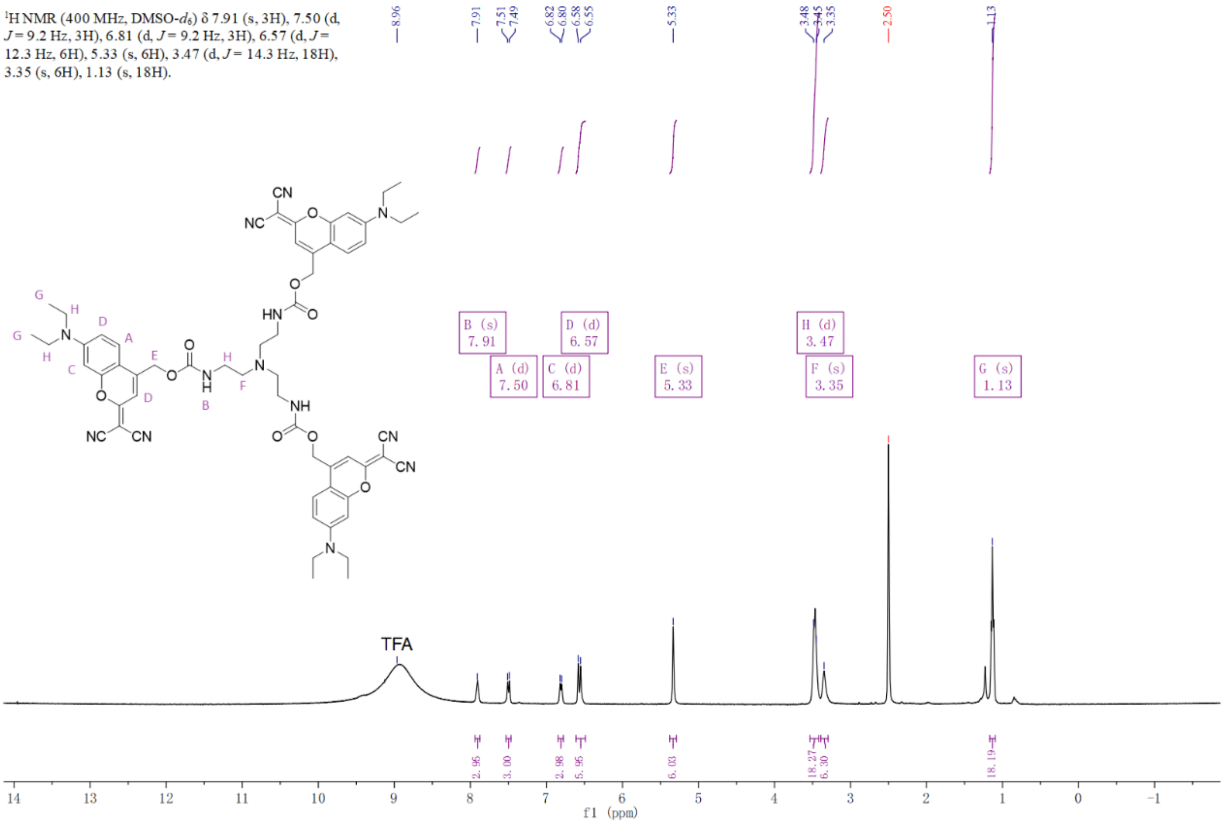

Figure S3.^{1}H-NMR spectrum of the DTAEA molecule with 1‰ trifluoroacetate (TFA) in DMSO-d_6.

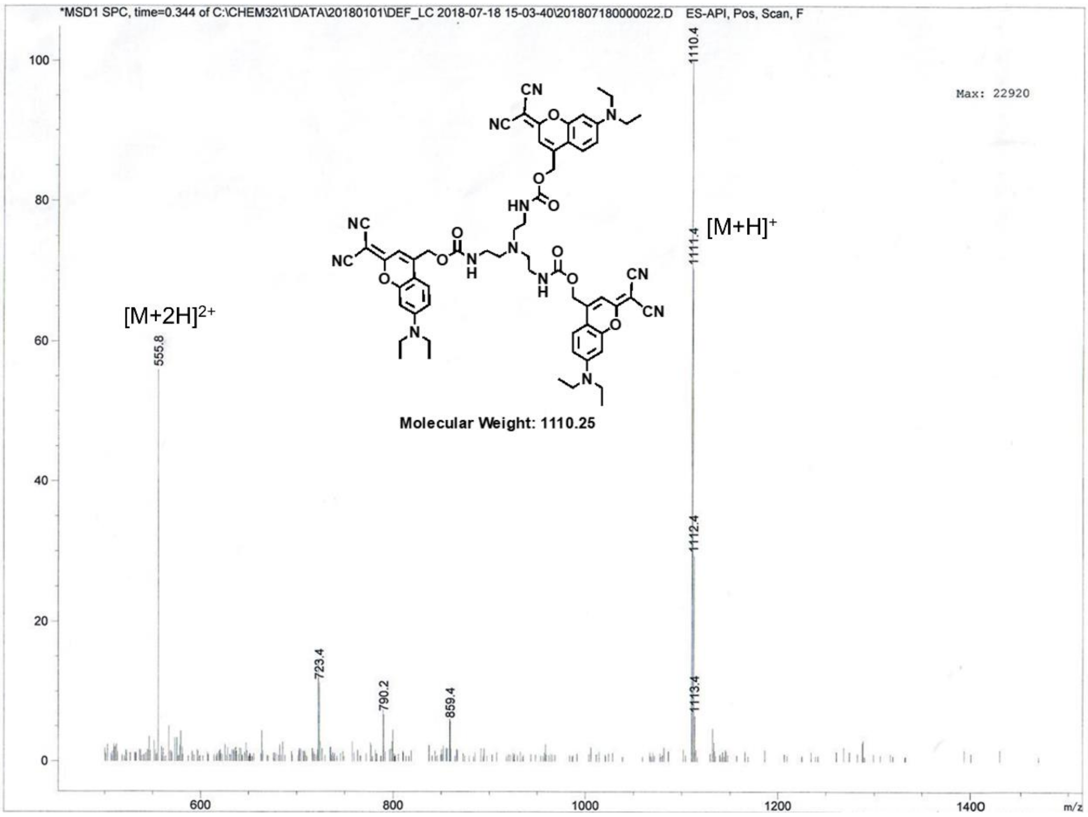

Figure S4. ESI-MS spectrum of the DTAEA molecule.

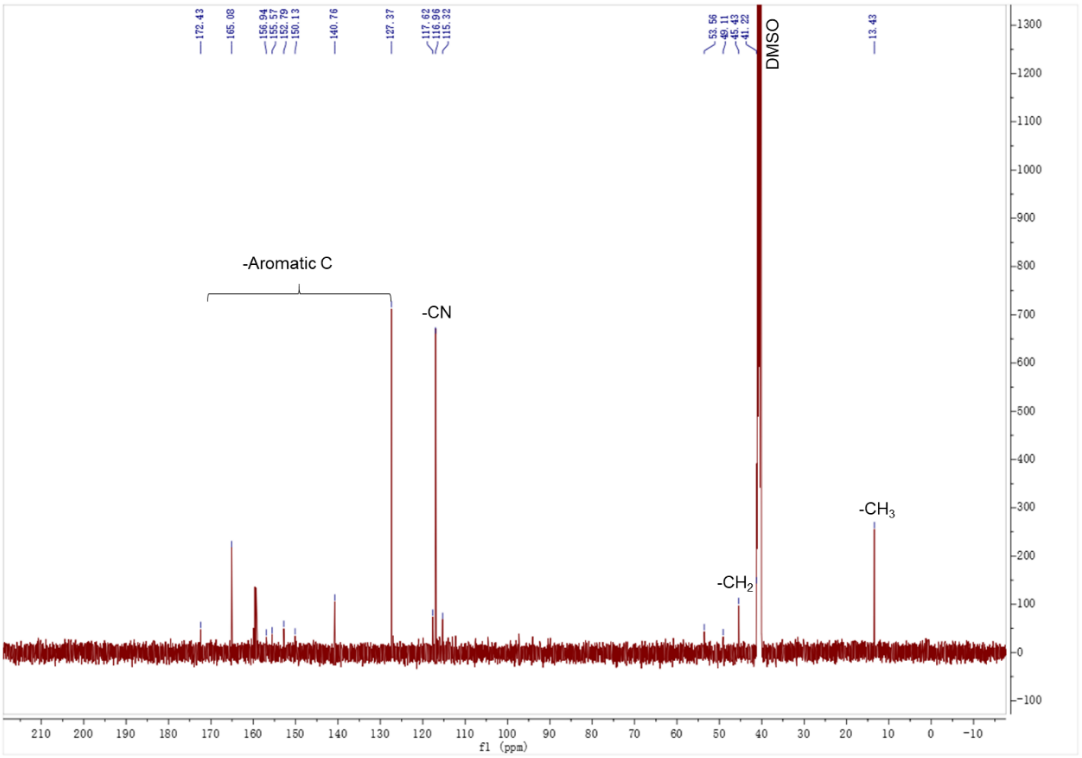

Figure S5. ^{13}C-NMR spectrum of the DTAEA molecule.

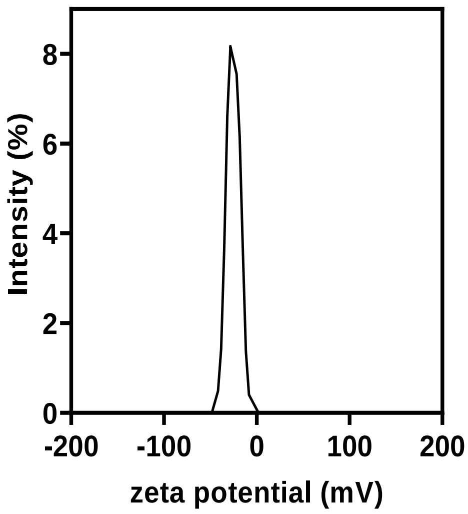

Figure S6. Zeta potential distribution of DTNPs in an aqueous solution.

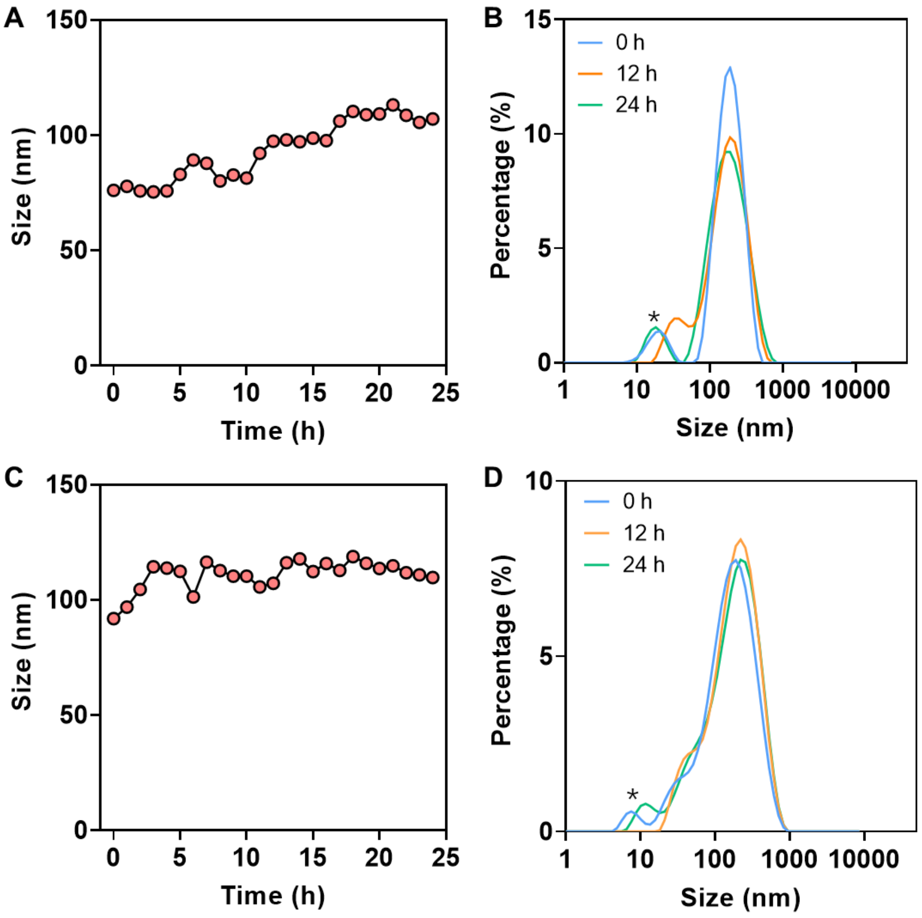

Figure S7. A) Size of DTNPs in 10% FBS-containing PBS within 24 h under 37 ºC. B) Size distribution of DTNPs in 10% FBS-containing PBS at 0 h, 12 h, and 24 h. C) Size of DTNPs in murine serum within 24 h under 37 ºC. D) Size distribution of DTNPs in murine serum at 0 h, 12 h, and 24 h. The star at around 10 nm labelled the intrinsic peak of the serum proteins.

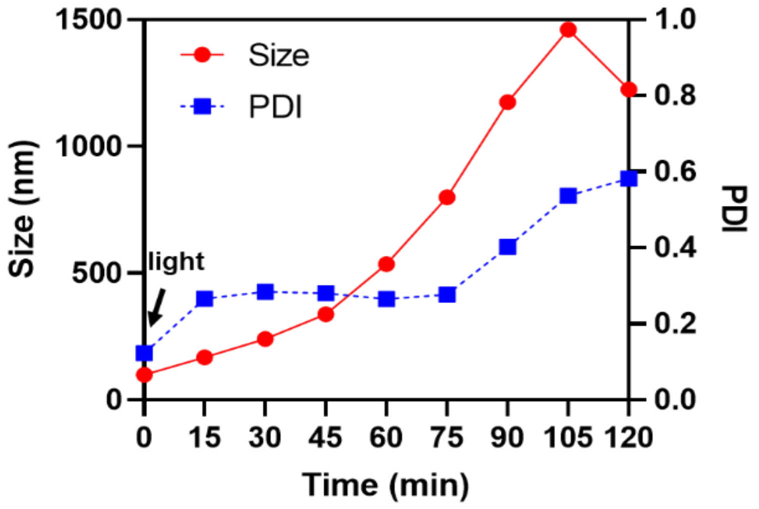

Figure S8. Size and PDI change of DOX/DTNPs within 2 h post-irradiation (505 nm, 50 mWcm^{-2}, 5 min).

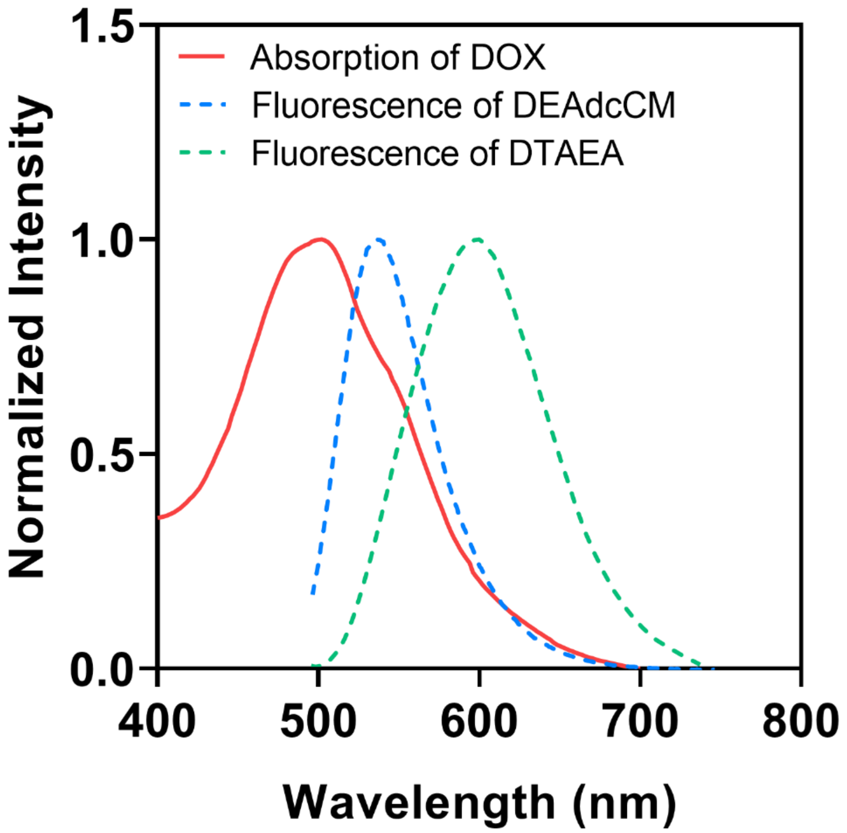

Figure S9. Normalized absorption spectrum (400–800 nm) of DOX and fluorescence emission spectra of DEAdcCM and DTAEA (Ex. 480 nm).

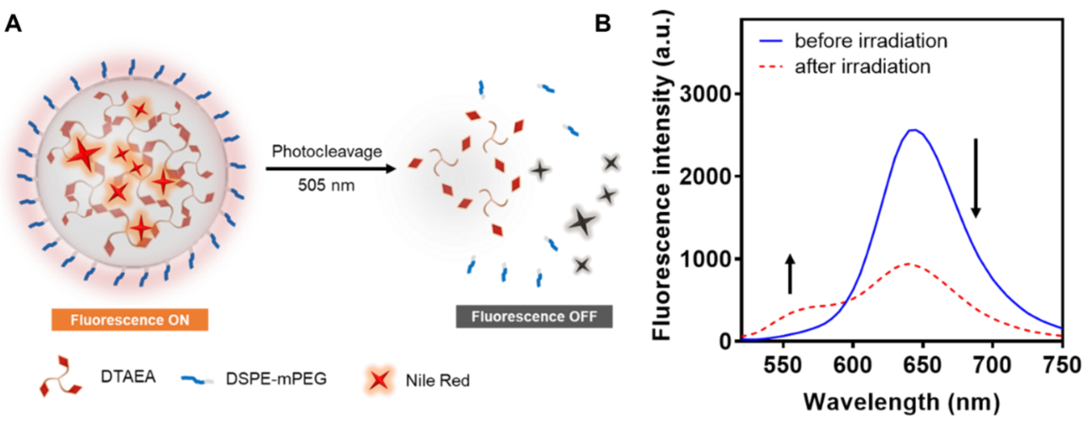

Figure S10. (A) Scheme of the proposed mechanism of fluorescence monitoring of light-triggered NR release from NR/DTNPs. (B) Fluorescence changes of the solution of NR/DTNPs after the light irradiation (505 nm, 50 mWcm^{-2}, 5 min).

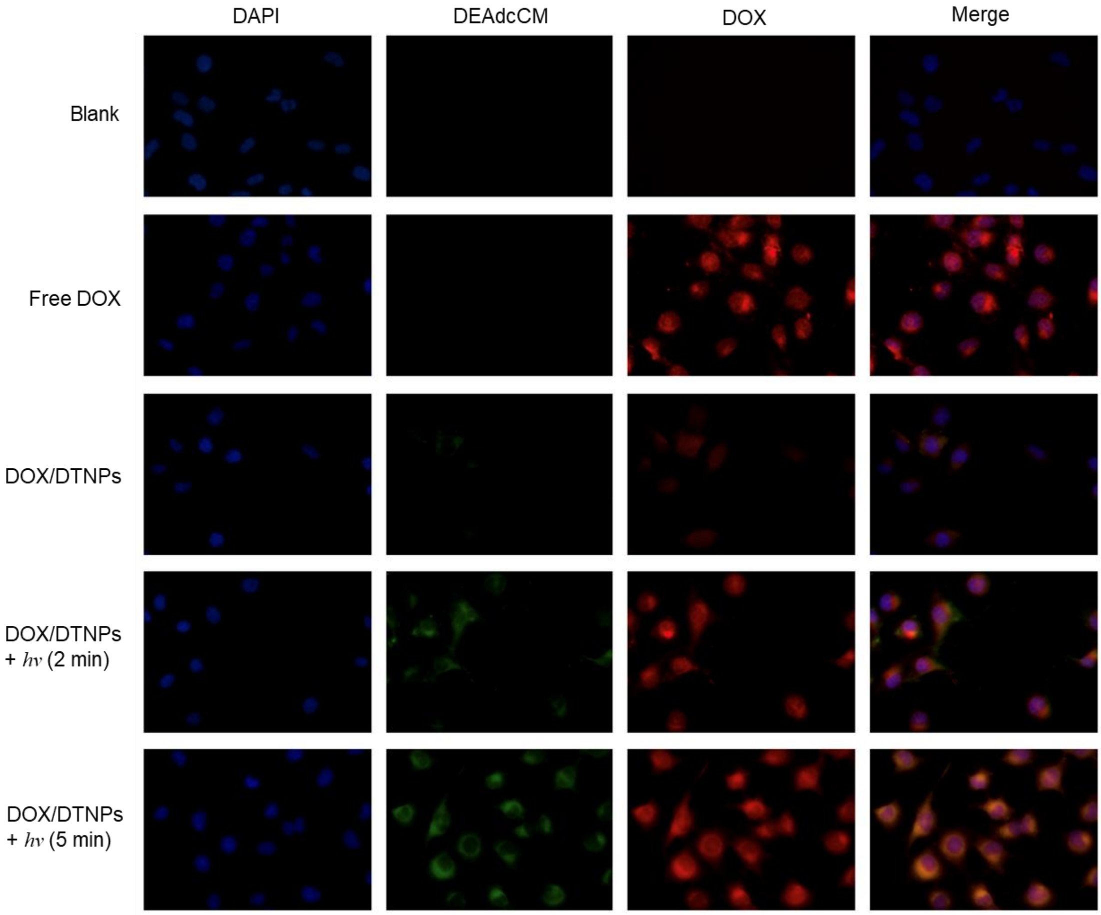

Figure S11. Representative fluorescence microscopic images of HUVEC cells after 2 h incubation with different formulations at an equivalent DOX concentration of 10 μM. The imageswereobtained under a 400X confocal microscope.

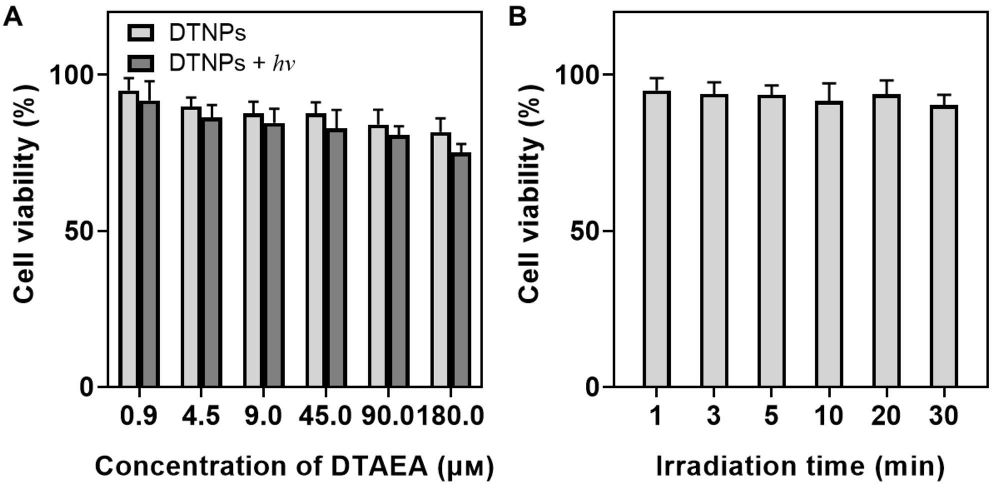

Figure S12. Cytotoxicity of DTNPs and light irradiation to HUVECs. (A) Cytotoxicity of DTNPs before and after thelight irradiation (505 nm, 50 mWcm^{-2}, 5 min). (B) Cytotoxicity of the light irradiation (505 nm, 50 mWcm^{-2}) for various time periods. Data were shown as means ± SD (n = 5).

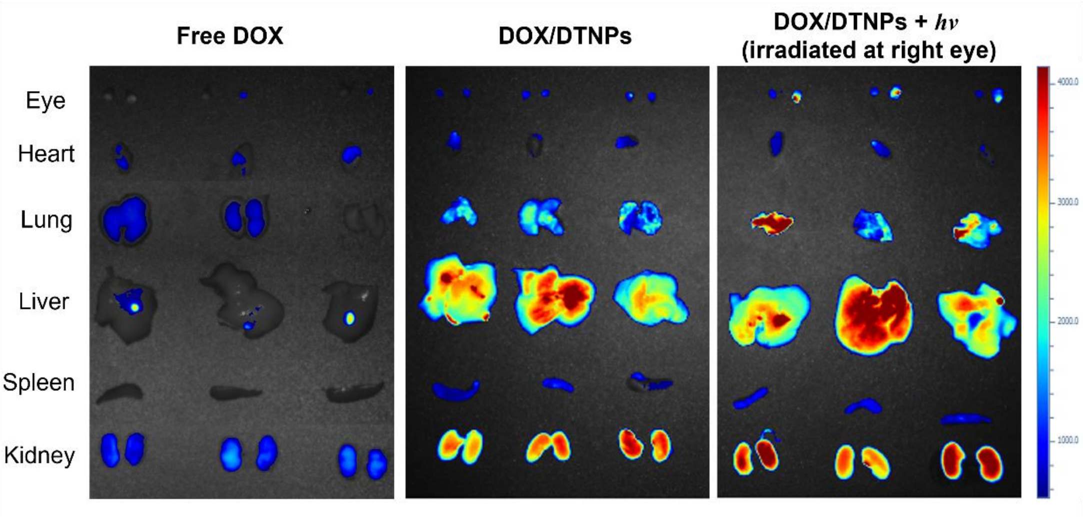

Figure S13. *Ex vivo* fluorescence imaging of the organs of retinoblastoma-bearing mice 1 h after the treatment of free DOX and DOX/DTNPs with or without light irradiation, separately (n = 3).

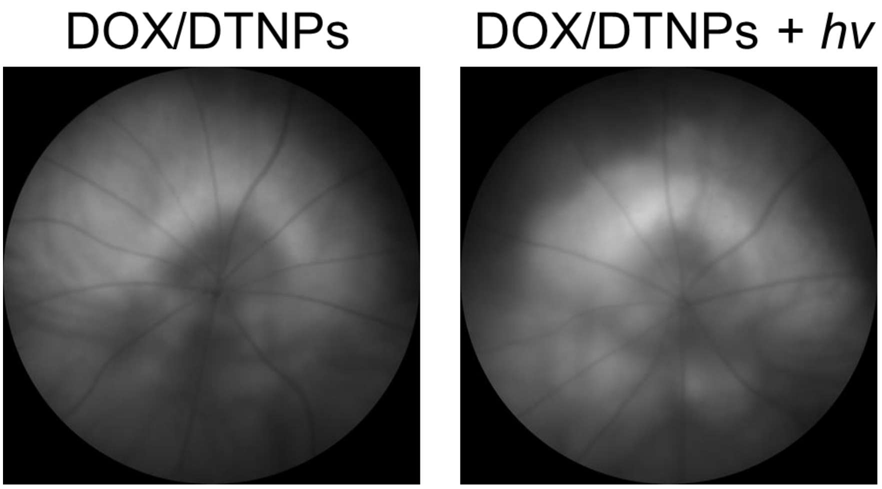

Figure S14. Murine fundus images of retinal blood vessels of the eyes treated with DOX/DTNPs and DOX/DTNPs + *hv*, separately. The dose of formulations was set as 5 $mgkg^{-1}$ on DOX basis. Light irradiation: 505 nm, 50 $mWcm^{-2}$, 5 min.

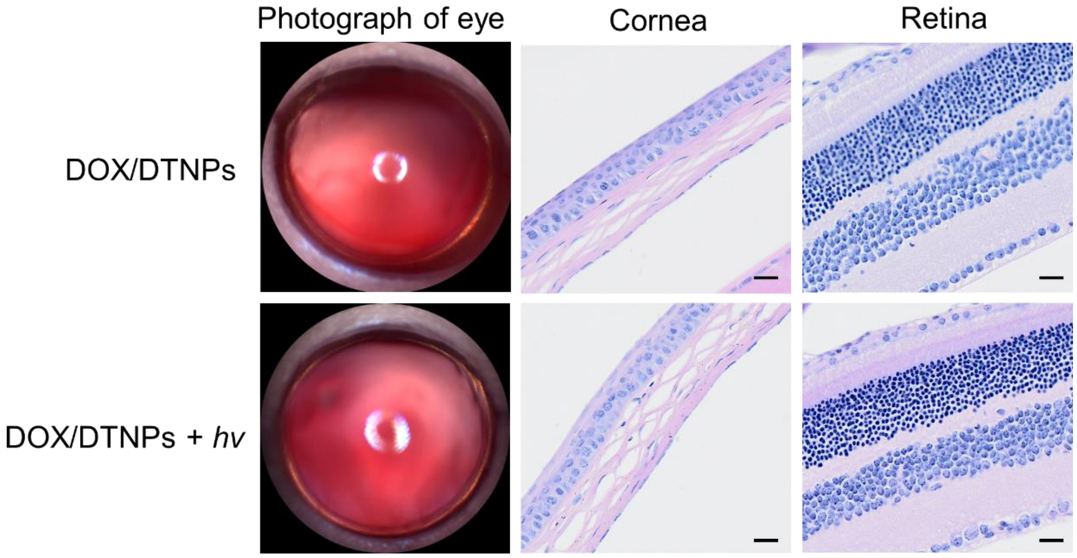

Figure S15. Murine ocular tissue reaction to DOX/DTNPs and 505 nm lightirradiation. Photographs showing clear cornea and lens, with an unobstructed view of retina through dilated pupil. H&E stained sections of cornea and retina, showing no detectable damage/injury. Scale bar: 20 μm.

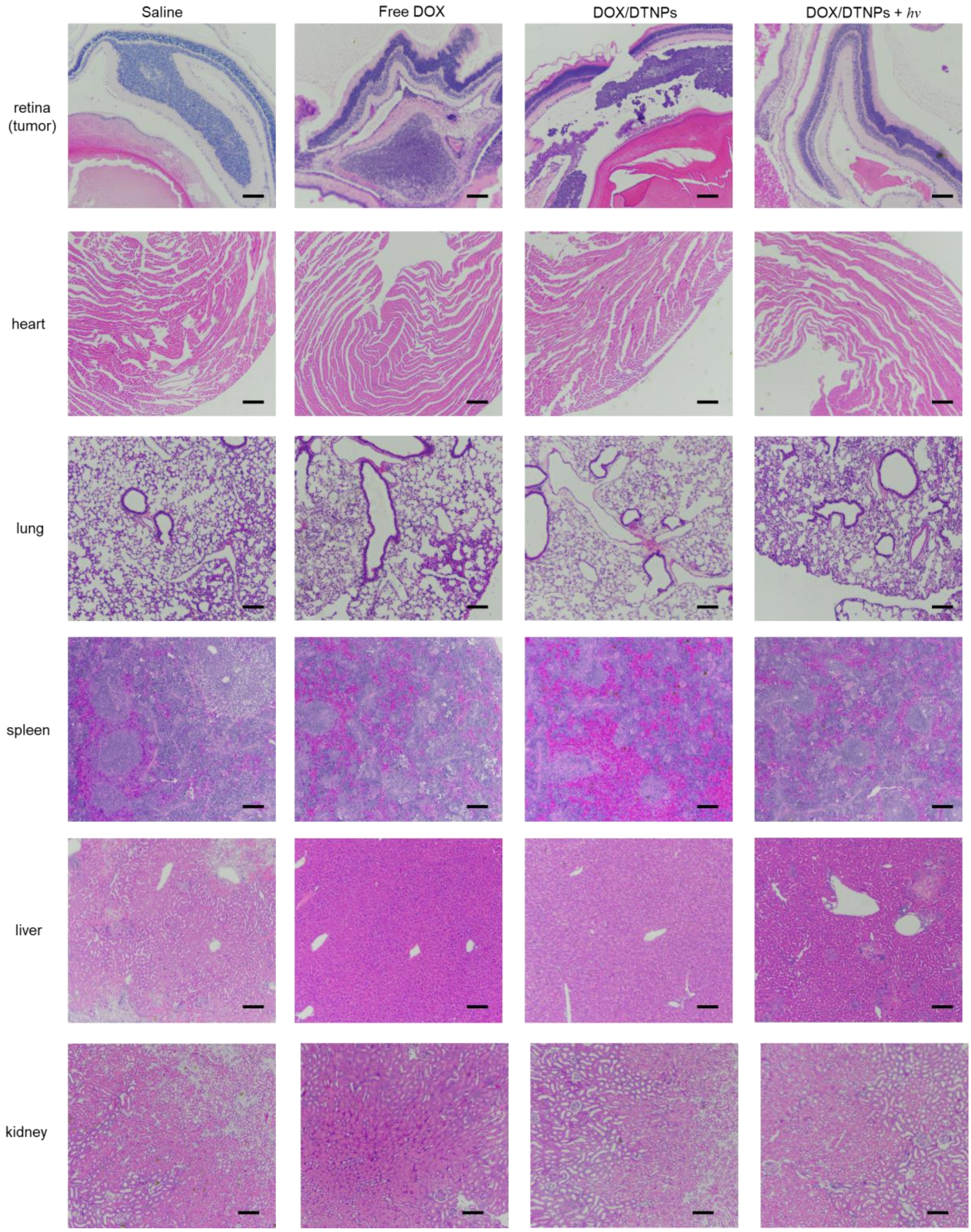

Figure S16. Representative photomicrographs of hematoxylin & eosin-stained sections of retina (with tumor), heart, lung, spleen, liver and kidney from four groups (saline, free DOX, DOX/DTNPs, and DOX/DTNPs + *hv*). Scale bar: 200 μm.

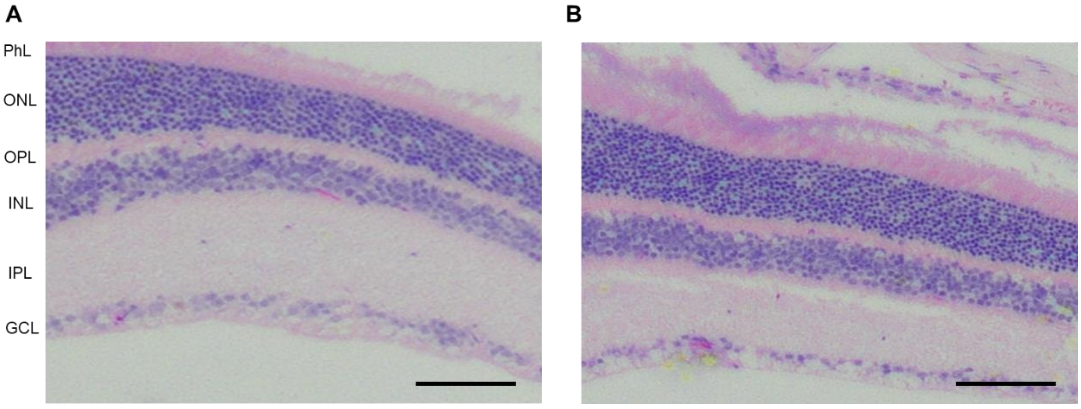

Figure S17. Representative photomicrographs of H&E sections of retina of the saline-treated group (A) and the group treated with DOX/DTNPs and the light irradiation (505 nm, 50 mWcm^{-2}, 5 min for 5 times in 12 days) (B). PhL, photoreceptor layer; ONL, outer nuclear layer; OPL, outer plexiform layer; INL, inner nuclear layer; IPL, inner plexiform layer; GCL, ganglion cell layer. Scale bar: 50 µm.

References

[1] K. Jiang, Y. Hu, X. Gao, C. Zhan, Y. Zhang, S. Yao, C. Xie, G. Wei, W. Lu, *Nano Lett.* **2019**, 19, 6410.

[2] W. Wang, Y. Chau, *Chem. Mater.* **2012**, 24, 946.

[3] W. Wang, Y. Chau, *Chem. Commun.* **2011**, 47, 10224.

[4] F. Zhang, X. Man, H. Yu, L. Liu, Y. Li, *Mol. Med. Report.* **2015**, 11, 1372.
